# Tissue Biomarkers in Melanoma Patients Treated with TIL

**DOI:** 10.1371/journal.pone.0048729

**Published:** 2012-12-20

**Authors:** Anne-Chantal Knol, Jean-Michel Nguyen, Marie-Christine Pandolfino, Gaëlle Quéreux, Anabelle Brocard, Lucie Peuvrel, Mélanie Saint-Jean, Soraya Saiagh, Amir Khammari, Brigitte Dréno

**Affiliations:** 1 INSERM, U892, CRCNA, Nantes, France; 2 Unité de Cancéro-Dermatologie-CIC biothérapie INSERM 0503, Nantes, France; 3 Unité de Thérapie Cellulaire et Génique, Nantes, France; 4 PIMESP-SEB, Hôpital St Jacques, Nantes, France; Université Paris Descartes, France

## Abstract

While treating stage III melanoma patients with autologous therapeutic TIL in an adjuvant setting, we previously reported a significant benefit of treatment on both progression-free survival and overall survival in patients with only one invaded lymph node (early stage III) compared to patients with more than one invaded lymph nodes (advanced stage III). In this context, in order to understand the difference of activity of TIL therapy according to the progression of the illness at stage III, the first objective of the present study was to determine potential differences in the characteristics of TIL populations obtained from an early stage III and a more advanced stage III when tumor burden is more important. The second objective was to determine possible differences in tissue expression level of several molecules involved in interactions between tumor cells and T cells between early and advanced stage III considering that the tumor microenvironment of invaded lymph nodes could become more tolerant with the progression of the disease. A total of 47 samples of melanoma invaded LN from stage IIIb (AJCC 2007) melanoma patients treated with TIL plus IL-2 were included in this study. We confirmed that both PFS and OS were significantly associated to the presence of tumor-reactive T-cells among TIL injected to the patients and that these tumor reactive T cells were more frequently observed at the early stage III. Moreover, while analyzing the expression of 17 markers on 34/47 tumor specimens using immunohistochemistry, we identified that 3 tissue markers involved in interactions between melanoma cells and T cells have a significant difference of expression between early and advanced stage III: MHC class I, adhesion molecule ICAM-1 and the co-stimulation molecule LFA-3 had a significantly weaker expression in melanoma tissue specimens from advanced stage III. In addition, the expression of the alpha chain of the IL-2 receptor (CD25) and the nuclear transcription factor Foxp3 was significantly increased in the melanoma tissue specimens from advanced stage III. Our results suggest differences in the immunological status of the tumor microenvironment between early and advanced stage III, which could explain the difference in clinical response to TIL infusion in an adjuvant setting between early and advanced stage III.

## Introduction

Metastatic melanoma prognosis remains poor and clinical success is still limited at this stage of the illness even if new treatments appear promising such as therapies targeting the CTLA-4 (cytotoxic T-lymphocyte associated antigen-4) or the mutated form of BRAF. Molecular markers that are able to predict patient prognosis after lymph node resection are therefore of clinical relevance. Independent prognostic factors could thus enable the identification of patients with a high risk of progression and who therefore would most benefit from adjuvant treatment. During the last decade, new insights in cellular and molecular biology have lead to the development of both active (vaccination) and adoptive immunotherapy protocols, the latter based on the transfer of high amounts of autologous expanded tumor infiltrating lymphocytes (TIL) extracted from the tumor tissue to melanoma patients [1]–[2]. The success of this immunotherapeutic approach would thus be directly related to the immunological status of the tumor microenvironment.

In advanced-stage (stage IV, AJCC, American Joint Committee on Cancer) melanoma patients, clinical trials using TIL combined with interleukin-2 (IL-2) have shown a response rate of 35% [3]–[4], and more recently, while using rapidly expanded tumor infiltrating lymphocyte cultures and high-dose IL-2 therapy after lymphodepleting conditioning, authors reported a 51% rate of objective clinical responses [5]. Another group reported that that lymphodepleting chemotherapy followed by transfer of short-term cultured TIL can mediate tumor regression in 50% of metastatic melanoma with manageable toxicity [6]. While treating stage III (AJCC 2007) melanoma patients with autologous therapeutic TIL, our group previously reported that both progression-free survival (PFS) and overall survival (OS) were significantly increased in the group of patients with only one invaded lymph node (early stage III) compared to the group of patients with more than one invaded lymph node (advanced stage III) [7]. With a long-term follow-up of 7 years, we confirmed that both PFS and OS were prolonged in the group of patients with early stage III (p-adjusted 0.0285) [8]. Moreover, while evaluating the presence of tumor-reactive T-cells among infused TIL in this 2 groups, we observed an absence of tumor-reactive TIL in advanced stage III only [9], and that the infusion of tumor-reactive TIL was statistically correlated with a prolonged PFS of early stage III patients [10].

In addition to a decrease in the number of tumor-reactive T-cells in several invaded lymph nodes, another hypothesis potentially explaining the difference of clinical results could be the development of in situ tumor escape mechanisms together with the progression of the illness. One of our challenges now is to identify which mechanisms could be involved in the development of this immune-tolerance between early and advanced stages. Melanoma tumor cells are discussed as cells able to induce an immune-tolerance in the tumor microenvironment. Tumor cells themselves may thus induce impairment of melanoma antigen presentation or secretion of immunosuppressive molecules such as IL-10 or TGF-β. Regulatory T-cells (Tregs) present in the tumor might also inhibit anti-tumor immune responses [11]–[12].

In this context, the first objective of the present study was to complete the analysis of TIL characteristics between early and advanced stage III melanoma (number of TIL, phenotype, proportion of tumor-reactive T-cells). The second objective was to determine possible differences in in-situ immunoreactivity of the tumor microenvironment between early and advanced stage III by studying the tissue expression level of several molecules involved in interactions between melanoma cells and T cells (tumor-associated antigens, major histocompatibility complex (MHC) molecules, cytokines, molecules implicated in T cell activation).

## Materials and Methods

### Melanoma samples

A total of 47 samples of melanoma invaded lymph nodes (LN) from stage IIIb (AJCC 2007) melanoma patients treated with TIL plus IL-2 were included in this study, corresponding to TNM classification T1-4b/N1a or N2a/M0 and T1-4a/N1b or N2b or N2c/M0 (AJCC 2010). Twenty-seven patients had only one invaded node (early stage III melanoma) and 20 patients had more than one invaded node (advanced stage III melanoma). The primary melanomas of these patients were all of the superficial spreading subtype (SSM) and of stage IIb or IIc corresponding to TNM classification T3b or T4a or T4b/N0/M0 (AJCC 2010).

After lymph node resection, one part of the invaded LN was used for producing therapeutic autologous TIL; another part was used for the immunohistochemical analysis and to establish the autologous tumor cell-line. The third part of invaded lymph node was processed for pathologic examination. Metastases were macrometastases. Written informed consent was provided by all patients and the study was approved by the Ethics Committee of Pays de La Loire (France).

### TIL production from the invaded LN

The same number of tumor fragments were placed in culture for both groups of patients. TIL were cultured according to a procedure previously described [2], [9]. Briefly, short term cultured TIL were isolated by culturing fragments of stage ΙΙΙ metastatic LN into 12-well tissue culture plates with X-VIVO 15 serum-free medium (BioWhittaker, Walkersville, MD, USA) containing 150 U/mL recombinant interleukin-2 (rIL-2) (Eurocetus, Rueil Malmaison, France) and glutamine (1 mM, BioWhittaker) for 10–14 days. *Ex vivo* expanded TIL were derived as follows: 1.8×10^6^ short term cultured TIL were plated at 300 viable lymphocytes/well with irradiated feeder cells (allogeneic peripheral blood leukocytes (PBL) and B-EBV cells: Epstein-Barr virus infected B-cells) into U-bottom microplates in 150 µL of rIL-2 medium. PHA-L (phytohemagglutinin-L or leucoagglutinin) (Difco, Detroit, ML, USA) was added on day 0 (1 µg/mL). Ten days later, lymphocytes were recovered from the culture plates, adjusted to 1×10^6^ cells/mL in rIL-2 medium and transferred into culture cell bags for an additional 10 days. The final TIL harvest was obtained by centrifuging, washing and suspending the TIL in 4% human serum albumin (LFB, Les Ulis, France). A second TIL expansion was performed within one month of the first, starting from cryopreserved short-term cultured TIL. Aliquots of TIL suspensions injected in the patients were cryopreserved to study their tumor specificity, which was carried out later once the autologous tumor cell line had been established in culture.

### Establishment of melanoma cell lines

Melanoma cell lines were established as previously described [13]–[14] and were successfully established for the 47 tumor samples.

Briefly, fresh LNs with metastasis were minced into small tumor pieces (approximately 1–2 mm^3^) with scissors and biopsy punch. The resultant fragment suspension was centrifuged and then pieces were inoculated (at a rate of 2 or 3 per well) in a 24-wells plate (NUNC) and 1.5 ml per well of RPMI (Roswell Park Memorial Institute) medium supplemented with 10% fetal calf serum (FCS) was added. Plates were placed at 37°C in a humidified incubator with 5% CO_2_ and observed under a light microscope every week and sub-cultured if necessary. The presence of tumor cells within established melanoma cell lines was assessed using both polymerase chain reaction (PCR) and flow cytometry based on the intracellular expression of tumor associated antigens (gp100, Melan-A/MART-1, tyrosinase, NY-ESO-1 and MAGE).

### Antibodies and flow cytometric analysis

The following antibodies were used: PE anti-CD2 (clone S5.2), PE anti-CD56 (clone MY31), PE anti-CD25 (clone 2A3), all from BD Pharmingen, Le Pont de Claix, France. We also used PC5 anti-CD3 (clone UCHT1), PE anti-CD8 (clone B9.11), APC anti-CD4 (clone 13B8.2), APC anti CD19 (clone J3-119), PC7 anti-CD45 (clone J.33), PE anti-CD16 (clone 3G8), all from Beckman Coulter, Marseille, France. Lymphocytes were gated according to their forward and size scatter characteristics, and FACSCanto analysis was performed using the BDFACS Diva software (BD Biosciences, San Jose, CA, USA).

### Cytokine production assay for evaluating the proportion of tumor-specific TIL

The fraction of tumor-reactive TIL was determined from the measurement of the fraction of interferon-gamma (IFN-γ)-secreting T cells among TIL stimulated by the autologous melanoma cell line, as described previously [9]. Briefly, about 1×10^5^ lymphocytes were stimulated by 3×10^5^ autologous melanoma cells in 200 µL of X-VIVO 15 medium in the presence of brefeldin A, 10 µg/ml (Sigma, St Louis MO, USA) in round-bottom 96-well plates. The cultures were incubated for 6 h at 37°C in 5% CO2 humidified atmosphere. Then cells were stained for surface markers with fluorochrome-labeled monoclonal antibodies (anti-human CD4 APC, anti-human CD8 FITC, BD Biosciences, France). For intracytoplasmic cytokine staining, cells were then fixed 10 min at room temperature in a solution of phosphate-buffered saline (PBS) 4% paraformaldehyde (Sigma), washed and stored at 4°C until labeling. Fixed stimulated lymphocytes were stained for cytokine production using the method described by Jung et al. [15]. Anti-IFN-γ specific antibody was purchased from BD Biosciences, France and diluted in PBS containing 0.1% bovine serum albumin (BSA) and 0.1% saponin (Sigma). After staining, cells were resuspended in PBS and 10^5^ events were analyzed on a FACScalibur flow cytometer using the BDFACS Cell Quest Pro software (BD Biosciences, San Jose, CA, USA). T cell responses were considered significant when the mean fluorescence labeling of TIL stimulated by the autologous tumor cell line exceeded, by at least half a log, the mean fluorescence of the background responses of non-stimulated TIL and/or of TIL stimulated by an HLA-mismatched melanoma line. A value of 0.3% was considered as the significance threshold.

### Immunohistochemistry on tumor LN

Immunohistochemistry (IHC) was performed using the streptavidin/peroxidase technique as previously described [16]. Deep-frozen sections were incubated for 30 minutes at room temperature with the primary antibody. 17 different monoclonal antibodies were used to explore the expression of tumor-associated antigens: anti-gp100 (clone HMB45, 8 µg/ml, Dako, Trappes, France), anti-Melan-A (clone A103, 8 µg/ml, Dako, Trappes, France), anti-tyrosinase (clone T311, 4 µg/ml, Novocastra); adhesion molecules: anti-ICAM-1 (CD54, clone 84H10, 5 µg/ml, Beckman Coulter), anti-LFA-3 (CD58, clone BRIC5, 10 µg/ml, AbSerotec); immunosuppressive cytokines: anti-IL-10 (clone B-S10, 2 µg/ml, Diaclone), anti-TGF-β (clone TB21, 2 µg/ml, AbSerotec); MHC molecules: anti-MHC class I (clone G46-2.6, 5 µg/ml, Becton Dickinson), anti-MHC class II (clone TU39, 5 µg/ml, Becton Dickinson); and T-cell associated molecules: anti-CD4 (clone MT310, 2.5 µg/ml, Dako), anti-CD25 (clone ACT1, 2 µg/ml, Dako), anti-Foxp3 (clone 236A/E7, 5 µg/ml, ebioscience, San Diego, California, USA), anti-PD-1 (clone J105, 5 µg/ml, ebioscience), anti-CD80 (clone MAB104, 5 µg/ml, Beckman Coulter), anti-CD86 (5 µg/ml, Lifespan), anti-PD-L1 (clone MIH1, 5 µg/ml, ebioscience), anti-PD-L2 (clone MIH18, 5 µg/ml, ebioscience). Negative controls were done using a mouse monoclonal immunoglobulin-G1 (IgG1) isotype control or a monoclonal immunoglobulin-G2a (IgG2a) isotype control (DakoCytomation). Slides were read with a Leica microscope (magnification×25). In each immunostained serial section, the entire tumour area was evaluated. Each score was evaluated on a five-point scale: an absence of expression, weak (1–25% of positive cells), moderate (26–50%), intermediate (51–75%) and strong expression (>75%) was represented respectively by levels 0, 1, 2, 3 and 4. Photographs were taken using a digital SLR Nikon D70S camera. To avoid the subjectivity of the reading, all the slides were read by two independent examiners in a blinded reading.

### Statistical analysis

Statistical analysis was performed using Wilcoxon test and t test to compare results from the group of patients with more than one invaded LN to results from the group with only one invaded LN. Correlation between the number of invaded LN and each parameter was assessed using Spearman correlation test.

Overall survival (OS) was defined as the time elapsed from the date of surgery to the date of death from any cause. Progression-free survival (PFS) was defined as the time elapsed from the date of surgery to the date of the first event. Survival curves were estimated using the Kaplan-Meier method. The Cox proportional hazard model was used for assessment of the predictive value of continuous parameters. R statistical software was used and statistical significance was set at p<0.05. No correction for multiple testing was applied in this exploratory study.

## Results

A total of 47 samples of melanoma invaded LN from stage IIIb (AJCC 2007) were included in this study. Mean age of the 47 patients from which melanoma samples were obtained was 55.1±11.3 years (median: 53; min.-max.: 34–75); sex ratio was 27 males for 20 females. For the 47 stage IIIb melanoma patients, median event-free survival was 26.2 months and median survival was 37.5 months.

PFS was confirmed significantly associated to the number of invaded nodes, being worse in the group of more than one invaded nodes (p = 0.00289). OS was also confirmed significantly associated to the number of invaded nodes, being worse in the group of more than one invaded nodes (p = 0.00496). The median survival duration without death or relapse was 5.25 months for the group of more than one invaded LN (advanced stage III) and 157.02 months for the group with only one invaded LN (early stage III).

### TIL obtained from the invaded LN

The 47 samples of melanoma invaded LN were used for producing therapeutic autologous TIL.

Amount of TIL produced between early and advanced stage III melanoma lymph nodes Patients with advanced stage III melanoma received significantly more therapeutic TIL (t test, p = 0.000585; Wilcoxon, p = 0.000539) (early stage III, 1^st^ expansion, mean 4.89×10^9^ TIL, 2^nd^ expansion, mean 4.5×10^9^ TIL; advanced stage III, 1^st^ expansion, mean 7.95×10^9^ TIL, 2^nd^ expansion, mean 10.22×10^9^ TIL). This result was confirmed using Spearman correlation test (p = 0.000319).

### Phenotype of TIL

There was no significant difference in the phenotype of expanded TIL infused to the patients between the two groups, regarding CD2-positive cells, CD3-positive cells, CD3/CD4 double positive cells, CD3/CD8 double positive cells or CD3/CD25 double positive cells (Table 1).

**Table 1:** Phenotype of large-scale expanded TIL from melanoma-invaded lymph nodes of 47 patients treated by TIL.

Patients	Relapse-free survival^c^	% CD2-positive^b^		% CD3-positive^b^		% CD3/CD4-positive^b^		% CD3/CD8-positive^b^		% CD3/CD25-positive^b^	
		E1^a^	E2	E1	E2	E1	E2	E1	E2	E1	E2
M88*	+	ND	ND	100	100	27	39	71	56	ND	19
M110	−(7)	100	99	99	99	78	84	18	18	51	40
M113	−(6.5)	100	100	99	100	36	75	63	28	9	42
M117	+	100	100	100	100	30	3	70	97	41	14
M125	−(3)	100	100	99	100	71	65	30	36	56	27
M131	−(5)	100	100	100	100	35	40	62	57	38	60
M132	−(2)	100	100	100	100	39	20	58	80	45	27
M134	+	100	100	100	100	55	62	45	38	25	22
M140	+	100	100	100	100	81	81	19	19	30	22
M153	−(3)	100	100	100	100	34	35	61	56	12	19
M154*	−(156)	100	100	100	100	59	63	40	39	12	17
M158	−(5)	100	100	100	100	30	31	70	63	9	16
M164	−(26)	100	100	100	100	60	61	38	39	28	14
M167	−(4)	100	100	100	100	24	18	74	79	15	12
M170*	+	100	100	100	100	30	32	67	67	16	36
M171	−(3)	100	100	100	100	48	42	52	58	19	11
M177*	+	100	100	100	100	42	63	56	32	34	24
M182	+	100	100	100	100	87	25	13	71	54	31
M187	+	100	100	100	100	16	24	86	76	5	10
M193	−(4)	100	100	100	100	14	27	86	73	7	15
M196*	−(6)	100	100	100	100	40	22	58	74	17	8
M197*	+	100	100	100	100	48	32	52	68	13	14
M199	−(2)	100	100	100	100	25	14	75	86	33	19
M200	−(7)	100	100	100	100	38	37	42	57	11	16
M204	−(3)	100	100	100	100	92	88	8	17	11	7
M212	−(8)	100	100	99	98	52	33	47	57	13	13
M218*	+	99	100	100	100	44	5	55	96	ND	ND
M234*	−(26)	100	100	100	100	62	49	42	51	ND	ND
M241*	−(13)	100	100	100	100	2	25	99	75	ND	ND
M245*	−(8)	100	100	100	100	32	26	68	74	ND	ND
M252*	−(2)	100	ND	100	ND	26	ND	71	ND	ND	ND
M254*	+	100	100	100	100	13	19	87	78	16	37
M263*	−(14)	100	100	100	100	22	30	77	68	11	5
M275*	−(8.5)	99.95	99.9	99.9	99.8	14.45	19.9	85.57	79.78	20.62	4.43
M278*	+	99.93	99.98	99.84	99.87	6.11	4.8	93.17	93.91	2.02	2.26
M284*	+	99.98	99.95	99.97	99.99	63.94	53.89	37.7	59.25	7.75	18.12
M288*	+	99.97	99.95	100	99.71	34.48	59.72	65.07	57.73	8.88	8.56
M301*	−(6)	99.93	99.99	99.74	99.92	24.43	30.64	72.44	66.83	2.06	3.22
M305*	+	99.98	99.92	99.93	100	19.1	20.08	80.9	79.99	6.22	2.97
M314*	−(3.5)	99.9	99.94	97.64	98.86	62.64	61.72	32	34.36	6.92	16.74
M329*	−(27)	99.86	100	98.63	99.45	34.65	39.97	62.88	57.22	16.2	13.09
M379*	−(4)	99.9	99.9	100	99.9	31.2	29.9	68.8	71	18.4	6.4
M386*	+	99.8	98.9	98.5	98.8	71.4	69.4	27.7	26.3	41.6	26
M415*	+	99.7	100	92	84.1	51.4	42.7	40.2	41.3	18.3	43.7
M417*	+	99.9	99.8	97.6	95.8	19	36.1	68.6	56.1	11.8	10.3
M424*	+	99.5	99.9	99.9	99.9	41.7	44	57.2	50.4	6.2	6.2
M428*	+	99.6	99.4	99.9	99.7	54.3	36.9	44.4	57.2	20.2	5.2

* Melanoma patients bearing only one metastatic lymph node (early stage III patients).

a E1 and E2 were TIL populations obtained and reinjected to the patient from respectively the first and the second ex-vivo expansions.

b Percentages of CD-positive TIL were estimated by membrane labeling. Cells were analyzed on a FACScalibur.

c Relapse-free survival of patients (−): patients who relapsed,(months); (+): patients who did not relapse.

### Cytokine production assay for evaluating the proportion of tumor-reactive TIL

**Figure 1:**
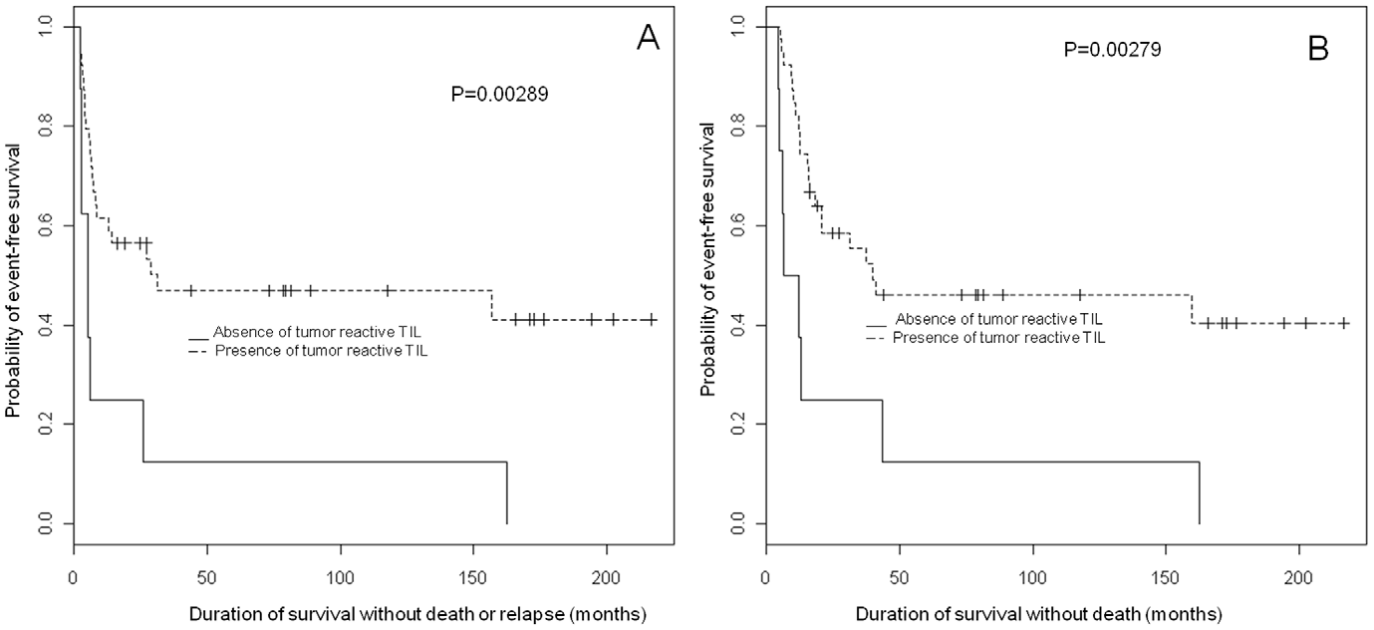
The presence of tumor-reactive TIL is associated to increased PFS and OS. PFS (A) and OS (B) curves for 47 patients from metastatic melanoma based on the presence of tumor-reactive TIL: cases were divided into two groups, presence of tumor-reactive TIL and absence of tumor-reactive TIL.

**Figure 2:**
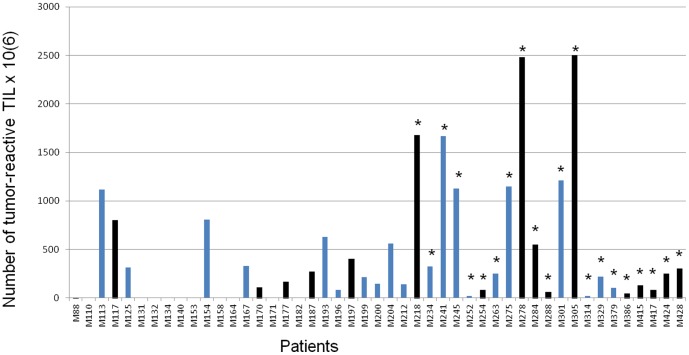
Number of tumor-reactive TIL injected in 47 patients. Black bars indicate relapse-free patients. * Indicates patients with only one melanoma-metastatic lymph node (early stage III patients).

The same 47 samples of melanoma invaded LN from which TIL were obtained, were used to evaluate the proportion of T cells reactive to the autologous melanoma cell line among TIL infused to the patients (Table 2, Figures 1 and 2).

**Table 2:** Fractions of tumor-reactive lymphocytes in large-scale expanded TIL from melanoma-invaded lymph nodes of 47 patients treated by TIL.

Patients	% IFN-γ-positive lymphocytes^b^		Relapse-free survival^c^
	E1^a^	E2	
M88*	ND	0.4	+
M110	0	0	−(7)
M113	10.3	0.8	−(6.5)
M117	ND	2.5	+
M125	0	2.4	−(3)
M131	0	0	−(5)
M132	0	0	−(2)
M134	0.7	0	+
M140	0	0	+
M153	0	0	−(3)
M154*	7.2	13.8	−(156)
M158	0	0	−(5)
M164	0	0	−(26)
M167	0.7	1.8	−(4)
M170*	1.2	ND	+
M171	0	0	−(3)
M177*	1.1	2.2	+
M182	0	0.7	+
M187	2.9	0.4	+
M193	3.5	4	−(4)
M196*	1	0	−(6)
M197*	2.9	0	+
M199	2.7	2.4	−(2)
M200	1	0.4	−(7)
M204	0	3.4	−(3)
M212	0.4	1	−(8)
M218*	9	11.3	+
M234*	4.9	2.3	−(26)
M241*	27.7	9.1	−(13)
M245*	19.7	11.3	−(8)
M252*	1.4	ND	−(2)
M254*	ND	2.2	+
M263*	2.56	1.49	−(14)
M275*	12.2	13	−(8.5)
M278*	21.5	11.6	+
M284*	2	4.7	+
M288*	0	1.5	+
M301*	13.2	14.7	−(6)
M305*	18.75	19.8	+
M314*	0.5	0.2	−(3.5)
M329*	3.4	4.2	−(27)
M379*	1.2	1.3	−(4)
M386*	1.1	0.9	+
M415*	5.8	2.2	+
M417*	0.56	0.89	+
M424*	ND	8	+
M428*	6.6	6.9	+

* Melanoma patients bearing only one metastatic lymph node (early stage III patients).

a E1 and E2 were TIL populations obtained and reinjected to the patient from respectively the first and the second ex-vivo expansions.

b Percentages of IFN-γ secreting TIL were estimated by intracellular labeling. TIL were stimulated 6 h by autologous melanoma cells in presence of brefeldin A. Then, cells were fixed, permeabilized, stained for cytokine production and analyzed on a FACScalibur.

c Relapse-free survival of patients (−): patients who relapsed,(months); (+): patients who did not relapse.

In the global population, we first observed that both PFS and OS were associated to the presence of tumor-reactive T-cells (Figure 1A and 1B): the duration of survival without death or without death or relapse, was increased by the injection of melanoma-reactive TIL (PFS: p = 0.00289; OS: p = 0.00279). The median survival duration without death or relapse was 31.4 months for patients that received tumor-reactive T-cells and 5.2 months for patients that did not receive tumor-reactive T-cells. Moreover, the presence of tumor-reactive TIL in the cellular expansion, was significantly associated to the stage of the illness number of invaded LN: 27/27 early stage III patients received melanoma-reactive TIL whereas 10/20 advanced stage III patients received melanoma-reactive TIL (t test, p = 0.000401; Wilcoxon, p = 0.000401): this result was confirmed using Spearman correlation test (p = 0.000401).

In addition, early stage III patients received significantly more specific TIL (1^st^ expansion: 6.89+/−7.87%, mean 0.37×10^9^, 2^nd^ expansion: 5.76+/−5.74%, mean 0.27×10^9^) than advanced stage III patients (1^st^ expansion: 1.17+/−1.47%, mean 0.11×10^9^, 2^nd^ expansion: 0.99+/−1.29%, mean 0.13×10^9^) (1^st^ expansion: t test, p = 0.004027; Wilcoxon, p = 0.000934, 2^nd^ expansion: t test, p = 0.000734; Wilcoxon, p = 0.000811) (Table 2, Figure 2). These results were confirmed using Spearman correlation test (1^st^ expansion: p = 0.000401, 2^nd^ expansion: p = 0.001414).

### Immunohistochemistry on tumor LN

A total of 34/47 tissue specimens of tumor invaded LNs from stage IIIb (AJCC) melanoma patients were available for protein expression using immunohistochemistry (21 early stage III and 13 advanced stage III). While analyzing a total of 17 markers, we found that the expression of 5 of them was significantly different between early stage III specimens and advanced stage III specimens.

**Figure 3:**
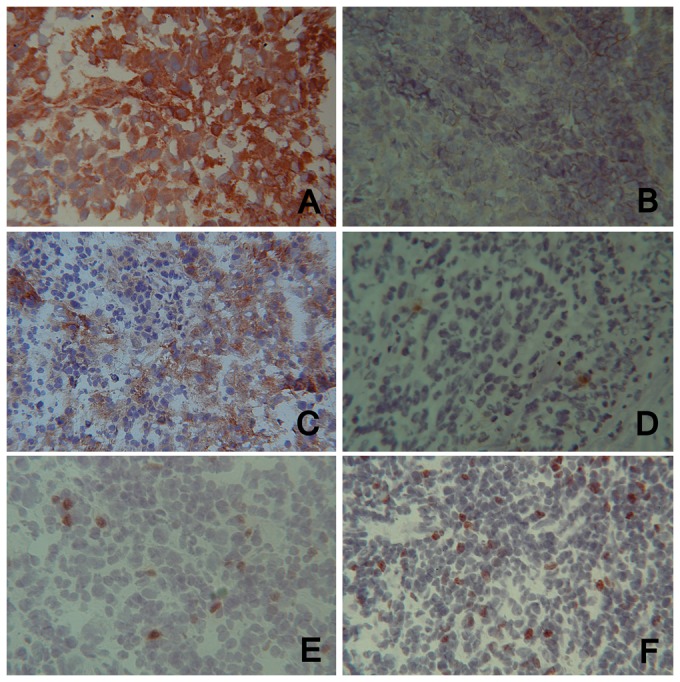
Representative pictures of MHC class I, ICAM-1 and Foxp3 expression using immunohistochemistry on tumor specimens of melanoma invaded LN. (A) and (B): MHC class I expression, (C) and (D): ICAM-1 expression, (E) and (F): Foxp3 expression (magnification×25). (A), (C) and (E) are specimens from the group with only one invaded LN whereas (B), (D) and (F) are specimens from the group with more than one invaded LN.

The expression of MHC class I molecules was significantly decreased in LN samples from advanced stage III patients (Figures 3A and 3B) (early stage III, mean expression: 2.8; advanced stage III, mean expression: 1.2; t test, p = 0.00002; Wilcoxon, p = 0.000132): this result was confirmed using Spearman correlation test (p = 0.0000342).

The expression of intercellular adhesion molecule-1 ICAM-1 (CD54) and co-stimulation molecule lymphocyte function-associated antigen 3 LFA-3 (CD58) was also significantly decreased in LN samples from advanced stage III patients (Figures 3C and 3D) (CD54: early stage III, mean expression: 2; advanced stage III, mean expression: 1.2; t test, p = 0.03715; Wilcoxon, p = 0.04918; CD58: early stage III, mean expression: 1.4; advanced stage III, mean expression: 0.3; t test, p = 0.0057; Wilcoxon, p = 0.00727): these results were confirmed using Spearman correlation test for CD58 only (p = 0.008).

On the contrary, the expression of the alpha chain of the IL-2 receptor (CD25) and of the nuclear transcription factor forkhead/winged helix transcription factor 3 (Foxp3) was significantly increased in LN samples from advanced stage III patients (Figures 3E and 3F) (CD25: early stage III, mean expression: 0.7; advanced stage III, mean expression: 1.2; t test, p = 0.0335; Wilcoxon, p = 0.04299; Foxp3: early stage III, mean expression: 0.2; advanced stage III, mean expression: 0.8; t test, p = 0.001626; Wilcoxon, p = 0.003021). These results were confirmed using Spearman correlation test (CD25, p = 0.02968; Foxp3, p = 0.000486).

The expression of the remaining 12 markers (gp100, Melan-A, tyrosinase, MHC class II, CD4, interleukin-10 (IL-10), transforming growth factor β (TGF-β), programmed cell death 1 (PD-1), programmed cell death ligands 1 and 2 (PD-L1 and PD-L2), CD80 and CD86) was not significantly different between the two groups.

## Discussion

In the present study, regarding the treatment, that consists to infuse ex-vivo expanded TIL to autologous melanoma patients, we first confirm our previously reported observation showing that both PFS and OS of patients treated with TIL were correlated with the presence of tumor-reactive T-cells among TIL infused to the patients [9]–[10].

In addition, we report here that despite patients in advanced stage III received a significantly higher amount of total TIL than patients in early stage III; early stage III patients received significantly more tumor-reactive TIL than advanced stage III patients.

Our results suggest that the pattern of infiltrating T lymphocytes in melanoma metastasis (from which TIL populations are obtained) in early stage III is different from that of advanced stage III. The earlier stages of the illness would thus be associated with a greater number of tumor-reactive T-cells that might explain that we obtain more specific T cells with a higher efficacy against melanoma cells within TIL expansions.

Melanoma patients usually display a spontaneous T cell response against their tumor, thanks to the presence of tumor-reactive T lymphocytes. However, at some point, the responder T cells become ineffective, probably because of a local immunosuppressive process occurring at the tumor sites [17]. Numerous immunomodulatory mechanisms have been described that either render tumor cells less sensitive to immune attack (resistance) or inhibit anti-tumor immune responses (immunosuppression). Considering that anti-tumor T cells are clearly capable of rallying to the tumor site, the spontaneous anti-tumoral T cell response must become ineffective either because the tumor cells have become insensitive to the effector cells or because the effector cells themselves have become unable to be stimulated by the antigen or to exert their function, a state referred as anergy by Boon et al [17]. Anergy may possibly be caused by tumor cells themselves, anergyzing directly the potential responder T cells, or by the generation of regulatory T cells.

In the present study, we then identified 5 tissue markers associated with a different level of in situ expression between early and advanced stage III which could potentially explain the difference of clinical response to the adoptive transfer of TIL between these two groups. The expression of MHC class I molecule, adhesion molecule ICAM-1 (CD54) and co-stimulation molecule LFA-3 (CD58) was significantly decreased in LN tumor samples from advanced stage III. In addition, the expression of the alpha chain of the IL-2 receptor (CD25) and the nuclear transcription factor Foxp3 which are notably expressed by Treg cells, was significantly increased in LN tissue from the group of advanced stage III.

The most common cause of resistance to immune cells is decreased antigenicity owing to MHC class I molecule down regulation or loss, which frequently occurs in human tumors [18]. In a previous work, we already reported a loss of expression for MHC class I molecule for 6 out of the 38 LN tissue specimens analyzed, all of these 6 samples coming from patients with more than one invaded LN [19], despite no statistical significance probably related to low number of patients. Recently, Yuan et al reported low to no expression of MHC class I and II molecules by all of the metastatic tumor samples of one patient pre vaccinated with gp100 and tyrosinase peptides and treated with anti-CTLA-4 Ipilimumab [20]. The absence or weak immune response against tyrosinase or gp100 was consistent with the recurrence of disease in that patient. Therefore, authors suggest that tumor escape from immune surveillance could be the consequence of the lack of expression of MHC class I and II molecules by the tumor tissue that would prevent effective antigen presentation to T cells. While studying 15 metastases from 2 vaccinated patients with melanoma [21], Carretero et al observed a differential activation of genes involved in inflammatory processes between regressing and progressing lesions obtained after immunotherapy and detected upregulation of genes related to antigen presentation in regressing lesions, particularly HLA expression [22]. Finally, authors propose that progressing lesions are not recognized by immune cells due to irreversible alterations in MHC class I molecules.

Regarding ICAM-1 and LFA-3, a weaker or absence of expression of these 2 molecules could contribute to a dysfunctional anti-tumor immune response in advanced melanoma, as suggested by the present results where tissue specimens from advanced stage III displayed weaker levels of ICAM-1 and LFA-3 expression. To our knowledge, the predictive value of ICAM-1 or LFA-3 associated with immunotherapy has never been previously investigated in human melanoma.

Although recent studies have questioned whether all Tregs are Foxp3+ or all Foxp3+ T cells are regulatory, Foxp3 remains the most specific marker of Tregs to date. Our group already recently reported that quantitation of Foxp3 expression using quantitative PCR appears as an independent prognostic factor for PFS in stage III melanoma patients with invaded lymph nodes [23]. We thus detected a significant reduced PFS in tissue specimens from patients with Foxp3^high^ expression compared with tissue specimens from patients with Foxp3^low^ expression. Thus, regulatory T cell populations represent a proportion of infiltrating cells and offer a potential for tumor-associated immune dysfunction. In a similar manner than our results, Hamid et al reported recently significant associations between clinical activity and high baseline expression of two immune-related genes, Foxp3 and indoleamine 2,3-dioxygenase (IDO) in advanced melanoma patients treated with anti-CTLA-4 Ipilimumab [24]. They also observed significant associations between clinical activity of Ipilimumab and increase in TIL between baseline and 3 weeks after beginning of treatment. Authors suggest thus that infiltrating lymphocytes may have anti-tumor activity and that changes in the immune microenvironment of the tumor may slow disease progression. In a pilot study on 15 patients treated with anti-CTLA-4 Tremelimumab, clinical response was also associated with increased tumor infiltration by CD8+ TIL and a variable association with CD4+ TIL [25]. In that study, the presence of Foxp3+ cells was variably associated with positive response. In the present work, we did not observe any difference in the expression of IL-10 or TGF-β between tissue specimens from early and advanced stage III patients. In a similar manner, no difference in the expression level of tumor-associated antigens was noted between the 2 groups. Moreover, we did not observe any difference in the expression of 5 additional markers that are relevant for the interaction of lymphocyte and tumor cells (PD-1, CD80, CD86, PD-L1 and PD-L2). These latest results suggest that there is no difference in the expression of some co-stimulatory and co-inhibitory molecules of the B7 family between early and advanced stage III, at least using an immunohistochemical analysis.

These results are consistent with the development of a growing immunosuppressive tumor microenvironment related to the tumor burden in melanoma. This immunosuppressive state would notably be induced by a decrease in the expression of molecules involved in interactions between tumor cells and T cells.

In this context, gene expression profiling in melanoma metastases has recently identified at least 2 broad subsets of tumors characterized by expression of immunomodulatory genes and specific subsets of immune effector cells [26]. While carrying out an open phase II trial of immunization with the recombinant MAGE-A3 protein in 72 patients with stage III–IV metastatic melanoma, a predictive gene signature (GS) that was associated with a significant improvement in the median OS was identified: 16.2 months in the GS(−) versus 28 months in the GS(+) population [27]. In particular, patients who responded clinically showed evidence of a high expression of immune-related cell markers.

In conclusion, in the present work, we report that in early stage III melanoma, a higher proportion of tumor-reactive T-cells among TIL infused to the patients is associated with an increase of both PFS and OS. We then identified 5 biomarkers that could play an important role in the efficacy of TIL infusion to melanoma patients. Therefore, the pattern of the host tumor microenvironment may emerge as a general predictor of possible clinical benefit in particular for treatment using TIL infusion.

## References

[1] Jotereau F, Labarriere N, Gervois N, Pandolfino MC, Dreno B (2003) [Passive immunotherapy of melanoma]. Bull Cancer 90: 583–586. 12957798

[2] Jotereau F, Pandolfino MC, Boudart D, Diez E, Dreno B, et al (1991) High-fold expansion of human cytotoxic T-lymphocytes specific for autologous melanoma cells for use in immunotherapy. J Immunother 10: 405–411. 1768674 10.1097/00002371-199112000-00003

[3] Rosenberg SA, Packard BS, Aebersold PM, Solomon D, Topalian SL, et al (1988) Use of tumor-infiltrating lymphocytes and interleukin-2 in the immunotherapy of patients with metastatic melanoma. A preliminary report. N Engl J Med 319: 1676–1680. 3264384 10.1056/NEJM198812223192527

[4] Rosenberg SA, Yannelli JR, Yang JC, Topalian SL, Schwartzentruber DJ, et al (1994) Treatment of patients with metastatic melanoma with autologous tumor-infiltrating lymphocytes and interleukin 2. J Natl Cancer Inst 86: 1159–1166. 8028037 10.1093/jnci/86.15.1159

[5] Dudley ME, Wunderlich JR, Yang JC, Sherry RM, Topalian SL, et al (2005) Adoptive cell transfer therapy following non-myeloablative but lymphodepleting chemotherapy for the treatment of patients with refractory metastatic melanoma. J Clin Oncol 23: 2346–2357. 15800326 10.1200/JCO.2005.00.240 PMC1475951

[6] Besser MJ, Shapira-Frommer R, Treves AJ, Zippel D, Itzhaki O, et al (2010) Clinical responses in a phase II study using adoptive transfer of short-term cultured tumor infiltration lymphocytes in metastatic melanoma patients. Clin Cancer Res 16: 2646–2655. 20406835 10.1158/1078-0432.CCR-10-0041

[7] Dreno B, Nguyen JM, Khammari A, Pandolfino MC, Tessier MH, et al (2002) Randomized trial of adoptive transfer of melanoma tumor-infiltrating lymphocytes as adjuvant therapy for stage III melanoma. Cancer Immunol Immunother 51: 539–546. 12384805 10.1007/s00262-002-0315-1 PMC11034217

[8] Khammari A, Nguyen JM, Pandolfino MC, Quereux G, Brocard A, et al (2007) Long-term follow-up of patients treated by adoptive transfer of melanoma tumor-infiltrating lymphocytes as adjuvant therapy for stage III melanoma. Cancer Immunol Immunother 56: 1853–1860. 17549472 10.1007/s00262-007-0340-1 PMC11030710

[9] Pandolfino MC, Labarriere N, Tessier MH, Cassidanius A, Bercegeay S, et al (2001) High-scale expansion of melanoma-reactive TIL by a polyclonal stimulus: predictability and relation with disease advancement. Cancer Immunol Immunother 50: 134–140. 11419180 10.1007/PL00006683 PMC11036836

[10] Labarriere N, Pandolfino MC, Gervois N, Khammari A, Tessier MH, et al (2002) Therapeutic efficacy of melanoma-reactive TIL injected in stage III melanoma patients. Cancer Immunol Immunother 51: 532–538. 12384804 10.1007/s00262-002-0313-3 PMC11032931

[11] Somasundaram R, Jacob L, Swoboda R, Caputo L, Song H, et al (2002) Inhibition of cytolytic T lymphocyte proliferation by autologous CD4+/CD25+ regulatory T cells in a colorectal carcinoma patient is mediated by transforming growth factor-beta. Cancer Res 62: 5267–5272. 12234995

[12] Terabe M, Berzofsky JA (2004) Immunoregulatory T cells in tumor immunity. Curr Opin Immunol 16: 157–162. 15023407 10.1016/j.coi.2004.01.010

[13] Gervois N, Heuze F, Diez E, Jotereau F (1990) Selective expansion of a specific anti-tumor CD8+ cytotoxic T lymphocyte clone in the bulk culture of tumor-infiltrating lymphocytes from a melanoma patient: cytotoxic activity and T cell receptor gene rearrangements. Eur J Immunol 20: 825–831. 1971794 10.1002/eji.1830200417

[14] Pandolfino MC, Saiagh S, Knol AC, Dreno B (2010) Comparison of three culture media for the establishment of melanoma cell lines. Cytotechnology 62: 403–412. 20730489 10.1007/s10616-010-9286-9 PMC2993863

[15] Jung T, Schauer U, Heusser C, Neumann C, Rieger C (1993) Detection of intracellular cytokines by flow cytometry. J Immunol Methods 159: 197–207. 8445253 10.1016/0022-1759(93)90158-4

[16] Chebassier N, El Houssein O, Viegas I, Dreno B (2004) In vitro induction of matrix metalloproteinase-2 and matrix metalloproteinase-9 expression in keratinocytes by boron and manganese. Exp Dermatol 13: 484–490. 15265012 10.1111/j.0906-6705.2004.00197.x

[17] Boon T, Coulie PG, Van den Eynde BJ, van der Bruggen P (2006) Human T cell responses against melanoma. Annu Rev Immunol 24: 175–208. 16551247 10.1146/annurev.immunol.24.021605.090733

[18] Garrido F, Cabrera T, Aptsiauri N (2010) “Hard” and “soft” lesions underlying the HLA class I alterations in cancer cells: implications for immunotherapy. Int J Cancer 127: 249–256. 20178101 10.1002/ijc.25270

[19] Quereux G, Pandolfino MC, Knol AC, Khammari A, Volteau C, et al (2007) Tissue prognostic markers for adoptive immunotherapy in melanoma. Eur J Dermatol 17: 295–301. 17540635 10.1684/ejd.2007.0203

[20] Yuan J, Ginsberg B, Page D, Li Y, Rasalan T, et al (2011) CTLA-4 blockade increases antigen-specific CD8(+) T cells in prevaccinated patients with melanoma: three cases. Cancer Immunol Immunother 60: 1137–1146. 21465316 10.1007/s00262-011-1011-9 PMC3654853

[21] Berd D (2004) M-Vax: an autologous, hapten-modified vaccine for human cancer. Expert Rev Vaccines 3: 521–527. 15485331 10.1586/14760584.3.5.521

[22] Carretero R, Wang E, Rodriguez AI, Reinboth J, Ascierto ML, et al (2011) Regression of melanoma metastases after immunotherapy is associated with activation of antigen presentation and interferon-mediated rejection genes. Int J Cancer 10.1002/ijc.26471 PMC3504975 21964766

[23] Knol AC, Nguyen JM, Quereux G, Brocard A, Khammari A, et al (2011) Prognostic value of tumor-infiltrating Foxp3+ T-cell subpopulations in metastatic melanoma. Exp Dermatol 20: 430–434. 21410773 10.1111/j.1600-0625.2011.01260.x

[24] Hamid O, Schmidt H, Nissan A, Ridolfi L, Aamdal S, et al (2011) A prospective phase II trial exploring the association between tumor microenvironment biomarkers and clinical activity of ipilimumab in advanced melanoma. J Transl Med 9: 204. 22123319 10.1186/1479-5876-9-204 PMC3239318

[25] Ribas A, Comin-Anduix B, Economou JS, Donahue TR, de la Rocha P, et al (2009) Intratumoral immune cell infiltrates, FoxP3, and indoleamine 2,3-dioxygenase in patients with melanoma undergoing CTLA4 blockade. Clin Cancer Res 15: 390–399. 19118070 10.1158/1078-0432.CCR-08-0783

[26] Harlin H, Meng Y, Peterson AC, Zha Y, Tretiakova M, et al (2009) Chemokine expression in melanoma metastases associated with CD8+ T-cell recruitment. Cancer Res 69: 3077–3085. 19293190 10.1158/0008-5472.CAN-08-2281 PMC3886718

[27] Gajewski TF, Louahed J, Brichard VG (2010) Gene signature in melanoma associated with clinical activity: a potential clue to unlock cancer immunotherapy. Cancer J 16: 399–403. 20693853 10.1097/PPO.0b013e3181eacbd8

